# Supplementary material for: β-Adrenergic Stimulation Synchronizes a Broad Spectrum of Action Potential Firing Rates of Cardiac Pacemaker Cells toward a Higher Population Average
Source: Cells. 2021 Aug 18;10(8):2124. doi: 10.3390/cells10082124 (PMC8391682; doi:10.3390/cells10082124)
Supplement: Supplementary file 1 [file cells-10-02124-s001.zip › Suppl_Figures_S1-S4.pdf]

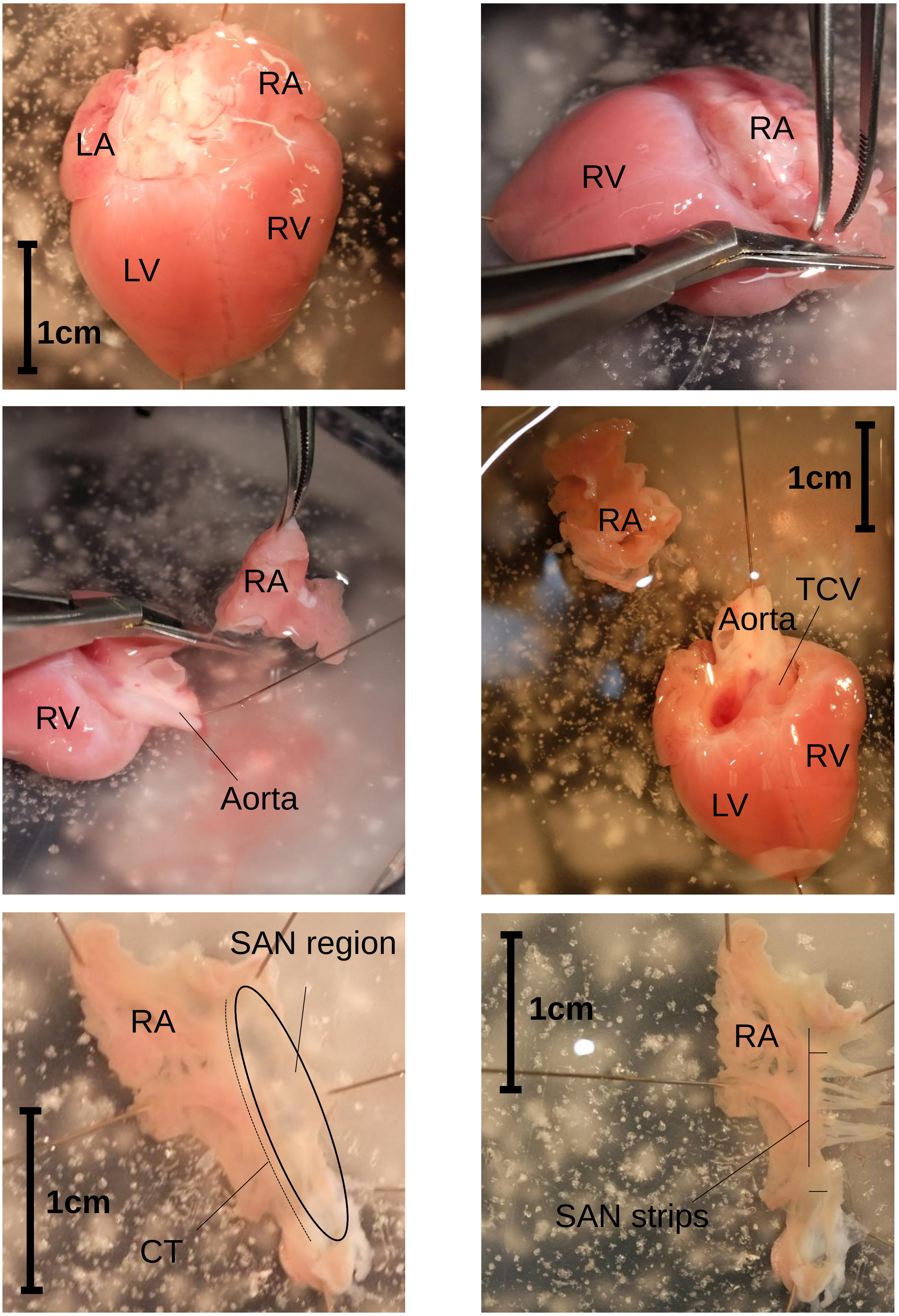

Figure S1: Location of guinea pig SA node in relation to the whole heart during our isolation procedure. LA=left atrium; LV=left ventricle; RA=right atrium; RV=right ventricle; TCV=tricuspid valve; CT=cristae terminalis; SAN=sinoatrial node. Modified from Kim et al. Cell calcium 2018, 74, 168-179, doi:10.1016/j.ceca.2018.07.002

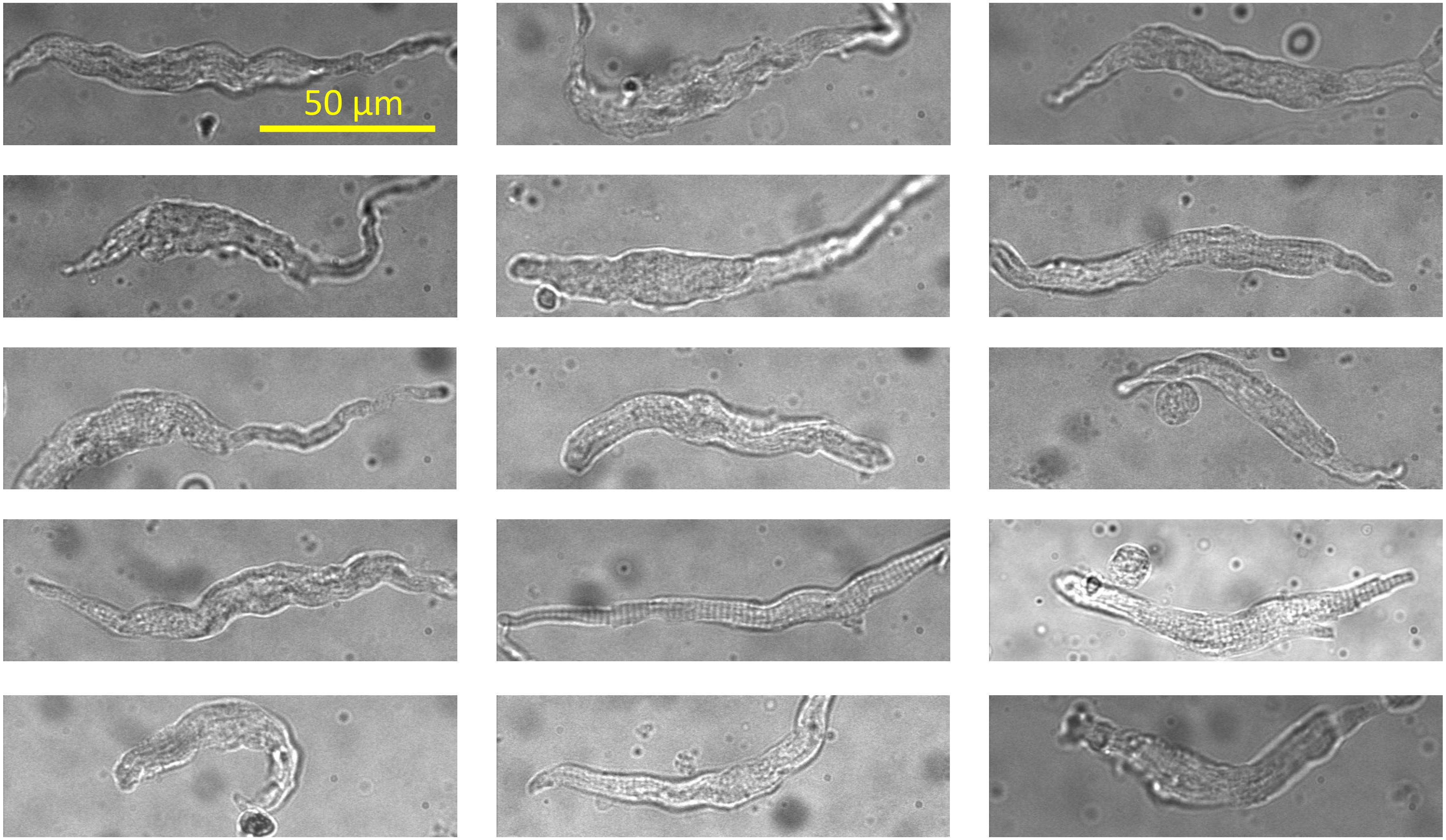

Figure S2: Typical morphologies of cells used in the present study.

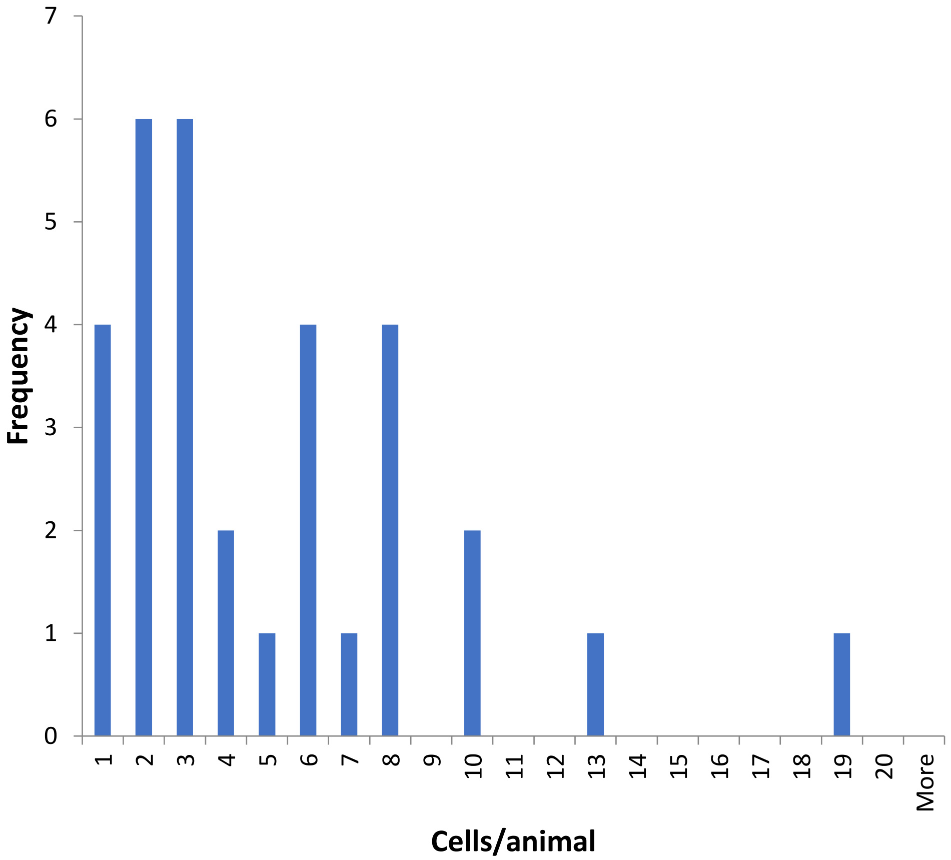

Figure S3: Distribution of our measurements in terms of cells/animal

A

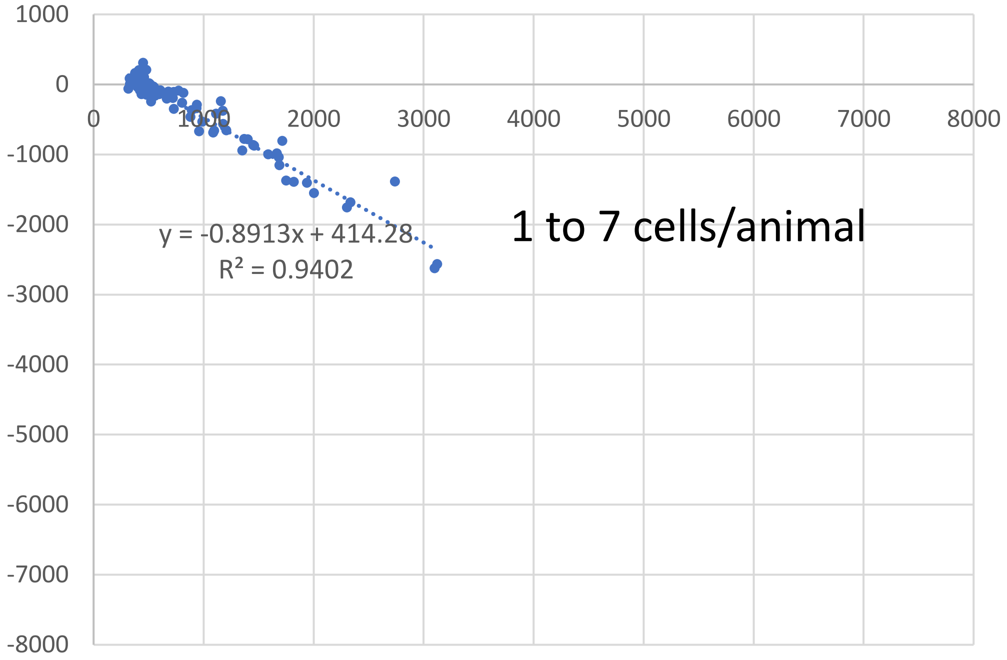

B

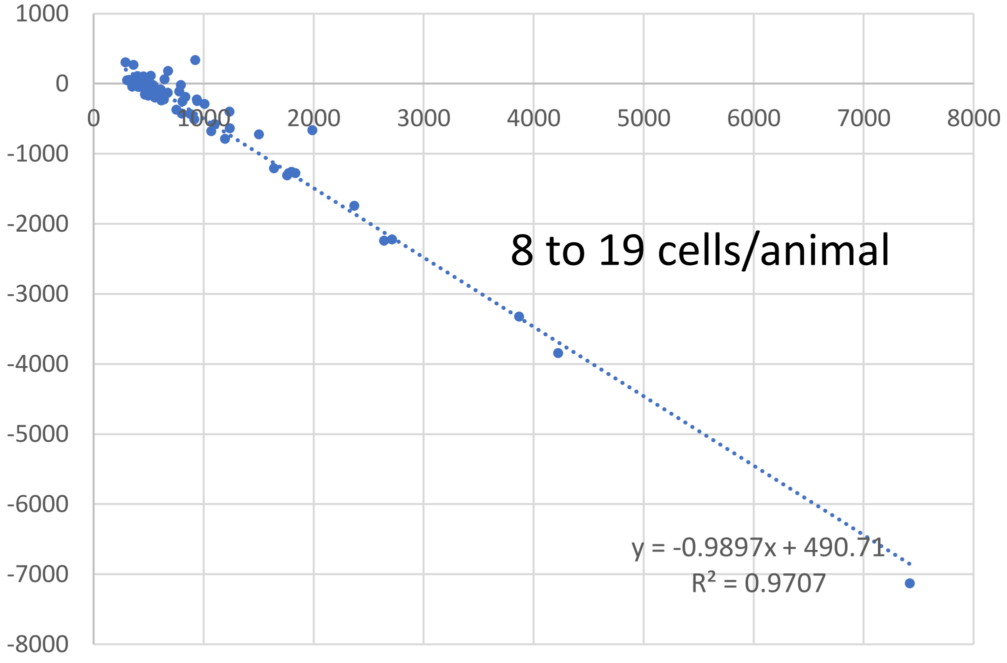

Figure S4: Results of linear regression analyses for subgroup of cells with different number cells measured per animal, illustrating robustness of our findings. (A): Analysis of 82 cells in the subgroup of 1 to 7 cells/animal. (B): Analysis of 84 cells in the subgroup of 8 to19 cells/animal. Regression line (dotted lines) equations are shown together with respective R^2 values.
