# Supplementary material for: β-Adrenergic Stimulation Synchronizes a Broad Spectrum of Action Potential Firing Rates of Cardiac Pacemaker Cells toward a Higher Population Average
Source: Cells. 2021 Aug 18;10(8):2124. doi: 10.3390/cells10082124 (PMC8391682; doi:10.3390/cells10082124)
Supplement: Supplementary file 1 [file cells-10-02124-s001.zip › Suppl_Table_S1.pdf]

Parameter	All cells		Cells/animal <=7		Cells/animal >=8	
Number of cells	166		82		84	
Conditions	Basal	ISO	Basal	ISO	Basal	ISO
Mean value	921.7614	507.3039	923.1902	514.636	920.3667	500.1464
SEM	66.47293	13.12734	71.66966	17.89898	111.6491	19.24943
SD	856.4438	169.134	648.9964	162.0821	1023.281	176.4239
CV%	92.91382	33.33977	70.29931	31.49452	111.1819	35.27445
Cells with unusual response (CL increase)	38 (22.9%)		17 (20.7%)		21(25%)	
Regression slope	-0.962		-0.8913		0.9897	
Regression offset	472.26		414.28		490.71	
R^2	0.961		0.9402		0.9707	

Table S1. Statistical data and results of linear regression analyses of AP cycle lengths (given in ms) measured in two subgroups of cells with different numbers of cells/animal compared to the results obtained in all cells. CL, cycle length of AP-induced Ca transient; ISO, isoproterenol; SD, standard deviation; SEM, standard error of mean; CV coefficient of variation (in %) = 100*Mean/SD.
